# Use of calcium during pregnancy: far beyond pre-eclampsia

**DOI:** 10.61622/rbgo/2025rbgo11

**Published:** 2025-03-17

**Authors:** Melania Maria Ramos Amorim, Marina Amorim Albuquerque, Lucas Félix, Anna Catharina Carneiro da Cunha, Leila Katz

**Affiliations:** 1 Instituto de Medicina Integral Prof. Fernando Figueira Recife PE Brazil Instituto de Medicina Integral Prof. Fernando Figueira, Recife, PE, Brazil.; 2 Universidade Federal de Campina Grande Campina Grande PB Brazil Universidade Federal de Campina Grande, Campina Grande, PB, Brazil.

Dear Editor,

We read with interest the Editorial of the Revista Brasileira de Ginecologia e Obstetrícia (RBGO) in which Braga et al. present the initiative of the state of Rio de Janeiro (Brazil) for prediction and secondary prevention of pre-eclampsia.^(1)^ The authors highlight universal calcium supplementation during pregnancy as a significant innovation, implemented for the first time on a population scale by a Brazilian state.

The Technical Note from the Rio de Janeiro State Health Department (SHD) published on 05/09/2024 defines local guidelines for preventing pre-eclampsia and, considering that all pregnant women in this state constitutes a population with low calcium intake and recommends supplementation with 1.5g/day of elemental calcium.^(2)^ This is an important initiative to be commendable and worthy of praise.

However, we were surprised reading the letter to the editor in which the authors provide their criticisms and considerations of the Rio de Janeiro SHD program.^(3)^ They question the recommendation for universal calcium prescription, claiming that there is no evidence supporting calcium supplementation for all pregnant women and citing the article published by Wright et al.,^(4)^ in which a sensitivity analysis of the Cochrane Systematic Review is carried out.

We understand that this controversy reflects international discussions regarding the use of calcium to prevent pre-eclampsia, which have intensified in recent months since the publication of the study of two clinical trials of calcium conducted by the WHO in India and Tanzania and published in the New England Journal of Medicine (NEJM) in January this year,^(5)^ involving 11,000 participants in each country, when was found that a low dose of daily calcium (500mg) was non-inferior to a high dose (1.5g) for pre-eclampsia, although non-inferiority was only demonstrated for prematurity in India, but not Tanzania.

Magee and von Dadelszen^(6)^ shortly thereafter published a letter to the Editor for the NEJM stating that the results of the two clinical trials of calcium would not be valid because as calcium does not work, it would not make sense to compare high doses and low doses, claiming that this result would be obvious given the lack of effectiveness of calcium in general. In the same issue of NEJM, Wright et al.^(7)^ make similar claims, citing their own study in which they perform the sensitivity analysis of the Cochrane Systematic Review and claiming that it is uncertain whether high-dose calcium actually reduces the risk of pre-eclampsia.

We regret that such discussions have reached the point of constituting true "Calcium Wars", the term we have called this intense debate about whether to supplement calcium for all pregnant women in countries with low calcium intake and so, would like to make our own comments.

Brazil is a country with a high frequency of pre-eclampsia (PE) and eclampsia.^(8)^ Hypertension is the main cause of maternal death in the country (20%), which results in more than 200 deaths from hypertension per year.^(8,9)^ In addition, our country is considered to have low calcium intake (below 900mg/day), according to data from IBGE.^(10)^ WHO in 2011, 2016 and 2018, recommend supplementing 1.5-2g/day of calcium during pregnancy in countries with low calcium intake^(9–11)^ and this recommendation is corroborated by the Rede Brasileira de Estudos da Hipertensão na Gravidez (RBEHG), in its most recent guideline on pre-eclampsia (2023).^(12)^

We highlight that this WHO recommendation was based on the very reliable Cochrane Systematic Review on calcium, latest version (2018).^(13)^ It is difficult to extrapolate to Brazil the recommendations and speculations that have been made by researchers from countries where calcium consumption is high and the incidence of pre-eclampsia is low, while our preeclampsia frequency is so high and our MMR has been stagnant for so many years (except for the increase during the COVID-19 pandemic). So that, we believe the effects of calcium certainly go far beyond pre-eclampsia.

We congratulate Professor Braga for his initiative at Rio de Janeiro SHD and we strongly suggest and wait for the WHO and RBEHG recommendations would be incorporated to the guidelines for prenatal care and hypertension in pregnancy in Brazil, so that the distribution and availability of calcium in Primary Health Care could be used by all pregnant women in the country.
